# Reregulated mitochondrial dysfunction reverses cisplatin resistance microenvironment in colorectal cancer

**DOI:** 10.1002/SMMD.20220013

**Published:** 2022-12-22

**Authors:** Yonghui Wang, Xiaodong Ma, Wenhui Zhou, Chang Liu, Hongbo Zhang

**Affiliations:** ^1^ Pharmaceutical Sciences Laboratory Åbo Akademi University Turku Finland; ^2^ Turku Bioscience Centre University of Turku and Åbo Akademi University Turku Finland

**Keywords:** biomimetic drug delivery, colorectal cancer, mitochondrial regulation, MSN‐based nanoparticles, multidrug resistance

## Abstract

Chemotherapy is one of the most basic and important treatments for malignant tumors. However, most chemotherapeutic drugs suffer from the resistance of tumor cells and lead to chemotherapy failure. Multidrug resistance (MDR) of tumor cells is the main obstacle to chemotherapy failure. The generation of MDR is not only the result of the performance of tumor cells, but the tumor microenvironment (TEM) also plays an important role in this process. The simultaneous dual intervention of cancer cells and the TEM has the potential to provide surprising results in overcoming MDR tumor therapy. Therefore, in this study, we designed a folate acid ligand‐modified nanoparticle (FA‐NPs) with a size of about 145 nm targeting multidrug‐resistant colorectal cancer and successfully co‐loaded cisplatin and Tris(2‐chloroisopropyl) phosphate (TCPP). FA‐NPs can enrich tumor sites through receptor‐mediated endocytosis. In vitro mechanism studies have shown that nanoparticles can reverse cisplatin resistance mainly by further increasing the level of reactive oxygen species in tumor cells, breaking the homeostasis of the internal environment, then trigging mitochondrial stress, regulating drug resistance‐related pathways, and improving the tumor drug resistance microenvironment; finally, the cisplatin recovers the antitumor effect with assistance from TCPP.

## Graphical abstract

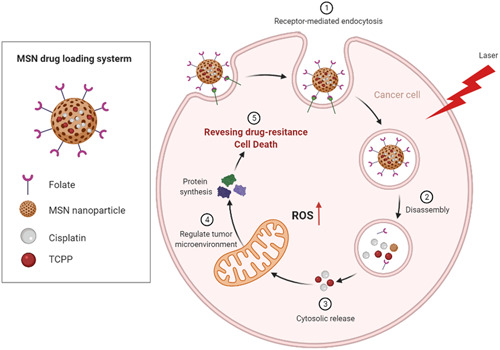

First, folic acid ligands were modified on the surface of MSN nanocarriers, and then TCPP and cisplatin were loaded simultaneously. When nanoparticles rely on receptor‐mediated endocytosis to accumulate in tumors, MSN will be slowly degraded under acidic conditions to release TCPP and cisplatin; then laser stimulation (0.2 W/cm^2^, 5 min) is given to trigger ROS generation, break the microenvironment balance, and finally reverse the resistance of HT‐29 cells to cisplatin through the regulation of mitochondrial stress and a series of signaling pathways.

1

**Key points**

- The authors prepared a bio‐mimic drug delivery system to reverse tumor drug resistance.
- Regulation of the tumor resistance microenvironment by disrupting the balance of reactive oxygen species in colon cancer cells.
- By modulating mitochondrial abnormalities, the drug resistance‐related protein levels are normalized which ultimately mediates tumor cell death.

## 2 INTRODUCTION

Colorectal cancer (CRC) is the third most common cancer type worldwide and threatens human health. In 2020, nearly two million new cases were diagnosed. It is the second most common cause of cancer death, killing nearly one million people each year.^1^ This fact remains despite the existence of effective screening techniques to reduce the number of deaths from the disease. Surgery combined with chemotherapy is a conventional treatment strategy for CRC, and significant progress has been made in the development of new chemo drugs for CRC treatments, for example, significantly improved survival in CRC stages III and IV, survival in patients with metastatic CRC has been extended to >20 months^2^, ^3^; however, CRC still incurable mainly due to the development of multidrug resistance (MDR) in tumor cells.

MDR is a major cause of chemotherapy failure, in which cancer cells simultaneously resist multiple chemotherapeutic agents that are structurally and functionally unrelated.^4^ Mechanisms of MDR include increased drug efflux, decreased drug uptake, impaired apoptotic pathways, and altered cell cycle checkpoints. Researchers developed countermeasures for each.^5^ Repeating history warns of a worrisome cycle: drug resistance—new generation drugs—drug resistance—new generation drugs—drug resistance. Are there strategies that are equally effective against different resistance mechanisms?

The tumor microenvironment (TEM) refers to the surrounding microenvironment of the tumor cells. TEM includes stromal cells (immune cells, fibroblasts, endothelial cells, microvessels, etc.) and the extracellular matrix.^6^, ^7^ High concentrations of reactive oxygen species (ROS) are often one of the characteristics of drug‐resistant cancer cells. For rapid growth and metabolism, cancer cells produce higher ROS than normal cells. Especially in drug‐resistant cancer cells that have oxidative stress caused by early treatment, the overall ROS level is even higher than normal cells, making it possible to break the homeostasis of ROS to kill drug‐resistant cancer cells.^8^, ^9^ Meanwhile, almost cancers are under the hypoxic microenvironment, and studies have shown that the hypoxic environment is significantly associated with the aggressiveness of tumors.^10^ In addition, many studies have shown that hypoxia will enhance drug resistance, increasing the difficulties of tumor treatment. There are a lot of mechanisms leading to hypoxia‐induced drug resistance, and all of them are multifactorial and complex. Recent studies found that hypoxia‐inducible factor alpha (HIF‐1α) is a vital factor in regulating hypoxia‐induced phenotypic changes, including drug resistance.^11^ More interestingly, there is increasing evidence that many intermediates also play an essential role in ROS‐mediated regulation of HIF‐1α.

A variety of physiological processes, such as adenosine triphosphate (ATP) production, apoptosis, and ROS generation cannot function well without mitochondria. Especially the mitochondrial respiratory chain is closely related to the regulation of apoptosis and ROS production. In mitochondrial respiration, oxygen is required throughout the entire process for the final transport of electrons. So during the process, oxygen (O_2_) levels decrease, electrons accumulate, and eventually the existing O_2_ molecules are monovalently reduced to superoxide (SO).^12^ Chandel et al.^13^ showed, under hypoxic conditions, that cancer cells tend to produce more ROS and stable HIF‐1α. Further evidence suggests that ROS has intimate connection with HIF‐1α. In particular, ROS produced by mitochondrial complex III are responsible for stabilizing HIF‐1α. Mitochondria have been proposed as viable targets for cancer therapy, disrupting the balance of mitochondrial homeostasis and perhaps seeking a general approach for the treatment of drug‐resistant cells.

Technological advances in drug delivery via nanotechnology have greatly impacted the medical field over the past few decades. More and more novel therapeutic and diagnostic platforms are emerging owing to the development of nano‐drug delivery technologies.^14^, ^15^ Nanocarriers for drug delivery have many wonderful advantages, including enhanced solubility of hydrophobic drugs, prolonged half‐life of drugs in blood, and better ability to target predetermined sites. But we are also concerned that nanoparticle delivery strategies relied on passive targeting with severe systemic side effects. So further developed nanoparticle technology focuses on active nanoparticle targeting, which is by introducing specific ligands bind to the surface of nanoparticles to exploit interactions with the target to further enhance drug delivery.^16^, ^17^ Such active targeting strategies enable the treatment and detection of diseases other than cancer in which enhanced permeability and retention effects (EPR) do not exist.

Therefore, in this work, we focused on TEM regulation by synthesizing nanoparticles to trigger the release of ROS to disrupt tumor homeostasis, then simultaneously targeting mitochondrial disorders to normalize them to reverse tumor resistance. This work is based on the mesoporous silica (MSN) nanomaterial; MSN has the characteristics of uniform mesoporous pore size continuously adjustable in the range of 2–50 nm, regular pores, stable framework structure, easy‐to‐modify inner and outer surfaces, and no physiological toxicity.^18^, ^19^ In addition, MSN has a huge specific surface area, which can slow down the release of drugs and improve the durability of drug efficacy. Therefore, in recent years, more and more attention has been paid to the application of MSN in controllable drug delivery systems.^20^ In this article, folate acid (FA) ligands were also modified on the surface of MSN, which can specifically target the highly expressed folate acid receptors on the surface of tumor cells, and simultaneously load Tris(2‐chloroisopropyl) phosphate (TCPP) and cisplatin to form FA‐NPs. TCPP can generate ROS under low‐intensity laser stimulation, break the ROS balance in the tumor‐resistant microenvironment, trigger a series of reactions, reverse the resistance of cisplatin, and exert its cytotoxic effect.

## 3 MATERIALS AND METHODS

### 3.1 Materials

Folic acid ligands were purchased from Shanghai Bide Pharmaceutical Co., Ltd. Phosphate buffered saline (PBS) and Bovine serum albumin (BSA) were purchased from Amresco. Dulbecco's modified Eagle's Medium (DMEM) cell culture medium, fetal bovine serum (FBS), and 0.25% trypsin‐EDTA were purchased from Gibco, Thermo Fisher Scientific. The Micro BCA protein assay kit was purchased from Bio‐RAD. The WST 1 assay from Abcam, Anti‐HIF‐1α antibody, anti‐glycogen synthase kinase‐3 beta (anti‐GSK‐3β) antibody, anti‐pyruvate kinase muscle isozyme (anti‐PKM2) antibody, and anti‐C‐Myc antibody, anti‐p53 antibody, anti‐phosphatase and tensin homolog (anti‐PTEN) antibody, and anti‐glyceraldehyde 3 phosphate dehydrogenase (anti‐GADPH) antibody were purchased from Novus Biologicals (R&D system). Triethanolamine (TEA), cetyltrimethylammonium chloride (CTAC), dimethylaminopyridine (DMAP), Carbodiimide hydrochloride (EDCI), aminopropyltriethoxy silane (APTES), and TCPP were purchased from MedChemExpress. ROS assay kit and single oxygen sensor green, SOSG, were from Abcam.

### 3.2 Methods

#### 3.2.1 Preparation of the MSN‐NP and FA‐NPs

For the preparation of MSN‐NPs, first, 0.18 g of TEA is weighed and mixed with 24 ml of (25 wt%) CTAC solution in a 100‐ml round bottom flask. This solution is put into the flask, and under continuous stirring 36 ml of water is added into it. Then, at 60°C to overnight formed water‐CTAC‐TEA solution, 20 ml of (10 v/v %) tetraethyl orthosilicate in cyclohexane is carefully added. Then the reaction system is maintained at 60°C with magnetic stirring for 24 h. After that, the products are collected by centrifugation and washed with ethanol several times to wash off the free chemical regents. 1 ml of MSN nanoparticles is taken, dried, and weighed to obtain the concentration of MSN and labelled and the solution is stored in ethanol.

Then, for FA targeting, FA ligand is used to bind to the surface of MSN‐NPs. First MSN‐NPs need to be activated by APTES in ethanol overnight to form MSN‐NH_2_. Then MSN‐NH_2_ is collected by centrifugation and the dichloromethane solution containing FA, DMAP, and EDCI is used to suspend them. This is incubated at room temperature and stirred overnight. The NPs are washed with excess dichloromethane. The MSN‐NP and FA‐NP are then stirred with TCPP and the cisplatin solution overnight to complete the drug loading. The free drug is removed by dialysis.

#### 3.2.2 Using transmission electron microscope to analyze the morphology of NPs

An appropriate amount of concentration of the MSN‐NP and FA‐NP was carefully added to a copper grid. After the samples were completely dried, they were observed under a transmission electron microscope.

#### 3.2.3 Characterization of the NPs

We used the Nanodrop to detect the drug encapsulation efficiency (EE) and drug‐loading (DL) capacity. For TCPP determination, a series of standard concentrations are set and the absorbance at 423 nm is measured. For cisplatin determination, in the same way, the absorbance at 301 nm is measured. We made standard curves for those two drugs first, as shown in Figure S1A,B, and the EE and DL were calculated by the following formula:

$$\text{EE}\;\left(\%\right)=\frac{\text{Weight}\;\text{of}\;\text{encapsulated}\;\text{drug}}{\text{Weight}\;\text{of}\;\text{drug}\;\text{added}}\times 100\%$$

$$\text{DL}\;\left(\%\right)=\frac{\text{Weight}\;\text{of}\;\text{encapsulated}\;\text{drug}}{\text{Weight}\;\text{of}\;\text{NPs}}\times 100\%$$

#### 3.2.4 In‐vitro drug release

Firstly, 1 mg of the NPs and FA‐NPs, respectively, is weighed. Then, 1.5 ml dialysis medium is used to suspend them. The release medium was 0.5% tween Milli‐Q (MQ) water (pH 5.4 and pH 7.4, respectively). Then they are put into tubes containing 50 ml dialysis medium. Finally, the whole set was placed in a constant temperature shaker with a rotational speed of 140 RPM/min (37°C). At different time points (0, 2, 4, 8, 16, 24, 36, and 72 h), 1 ml dialysis medium is used as sample and the same amount of refresh dialysis medium is replenished. The samples are diluted 100 times and measured by Nanodrop. Based on the standard curve, the cumulative release of TCPP and cisplatin is calculated.

#### 3.2.5 Cell culture

The HT‐29 cell line was cultured in Dulbecco's modified Eagle's medium (DMEM) with 10% FBS and 1% antibiotics of streptomycin and penicillin and then cultured at 37°C, 5% CO_2_.

The MCF‐10A cell line was cultured in Dulbecco's Modified Eagle Medium: Nutrient Mixture F‐12 (DMEM/F12) with 5% FBS, 10 μg/ml insulin, 20 ng/ml epidermal growth factor, 0.5 mg/ml hydrocortisone, 100 ng/ml cholera toxin and 1% antibiotics of streptomycin and penicillin, and then cultured at 37°C, 5% CO_2_.

#### 3.2.6 In vitro cytotoxicity

HT‐29 cells are passaged and collated and then seeded in 96‐well plates with a density of 5 × 10^3^ cells per well and incubated for 24 h. The different groups are set, group 1: free cisplatin, group 2: TCPP, group 3: cisplatin and TCPP (cisplatin + TCPP), group 4: MSN‐NP without drug load group (MSN) and 4: MSN‐NP with drug load group (NP), group 5: FA‐NP group, respectively. From group 2 to group 5, also two different treatments are set, which are without and with laser treatment, in which the laser groups are named as TCPP + L, cisplatin + TCPP + L, NP + L, and FA‐NP + L. The concentrations of cisplatin were set at 0, 1, 2, 4, 8, 10, 12, 16, and 20 µg/ml. For laser groups, gave we cultured with drugs for 24 h, and changed the fresh culture medium, gave the 650 laser (0.2 W/cm^2^, 5 min), and continued to culture for 24 h. For the groups without laser treatment, we continued to culture with drugs for 48 h. After 48 h, WST‐1 is used to measure cell viability.

#### 3.2.7 Cell uptake

Firstly, HT‐29 cells were seeded in a 6‐well plate with a cell density of 5 × 10^6^ per well overnight. Afterward, we added a culture medium containing NPs with or without FA ligand into the plate, respectively. Then, the cells are collected up to the time points and then tested under the flow cytometry.

#### 3.2.8 Calreticulin exposure

Firstly, HT‐29 cells were seeded in a 6‐well plate with a cell density of 5 × 10^6^ per well overnight; then different groups were set up, namely blank, cisplatin, TCPP, Cisplatin + TCPP, NPs, and FA‐NP groups. Except blank and cisplatin groups, other groups were divided into laser group and non‐laser group; the intensity of laser is 0.2 W/cm^2^. An amount of 6 µg/ml concentration of cisplatin and TCPP was added to each group, and the concentration of nanoparticles was calculated according to the same amount of cisplatin, after 24 h of co‐culture. In the laser group, the drug‐containing medium was removed and the fresh medium was added and the laser was given to irradiate for 5 min. Then, the culture was continued for 24 h. Cells were fixed with 80% methanol and permeabilized with 0.1% PBS‐Triton X‐100. Antibodies were then diluted to a final concentration of 0.1 µg/ml and detected by flow cytometry after 30 min incubation.

For confocal experiments, cells were seeded in confocal dishes at a density of 1 × 10^5^, and cells were dosed in the same manner as flow cytometry. Cells were fixed with 80% methanol and permeabilized with 0.1% PBS‐Triton X‐100 and then incubated antibody for 30 min at +4°C. The nuclear DNA was then incubated with DAPI for 10 min, and images were taken with the laser scanning microscopy 880 (LSM‐880) instrument.

#### 3.2.9 HMGB1release

Firstly, HT‐29 cells were seeded in a 6‐well plate with a cell density of 5 × 10^6^ per well overnight; group settings and drug treatment are the same as in Section 2.2.8. The culture was continued for 24 h. Cells were fixed with 4% formaldehyde for 10 min and then permeabilized with 0.1% PBS‐Tween for 20 min. Antibodies were then diluted 1/50 and incubated with samples for 30 min before detection by flow cytometry.

For confocal experiments, cells were seeded in confocal dishes at a density of 1 × 10^5^, and they were dosed in the same manner as flow cytometry. Cells were fixed with 4% formaldehyde and permeabilized with 0.1% PBS‐Triton X‐100, and then incubated with 1/500 dilution of antibody for 30 min. The nuclear DNA was then incubated with DAPI for 10 min before images were taken with the LSM‐880 instrument.

#### 3.2.10 Detection of singlet oxygen by NPs in vitro

The reagents were first prepared as stock solutions of approximately 5 mM in methanol by adding 33 µL of methanol to each 100 µg vial. The working solution of this reagent is prepared immediately before use and then a certain concentration of TCPP and NPs is removed with the same concentration, and immediately Varian scan (Ex/Em: 504/525 nm) is used to detect the optical density (OD) value at different time points after irradiating the laser for 30 s and the corresponding curve is drawn.

#### 3.2.11 Intracellular ROS detection

Firstly, HT‐29 cells were seeded in a 6‐well plate with a cell density of 1 × 10^5^ per well overnight. The cell administration method was similar to the above operation, and an additional group of positive control was added. After 24 h, DCFH‐DA was diluted 1:1000 with serum‐free medium to make the final concentration of 10 μmol/L. Except for the negative control, all other groups were added with probes and incubated in a 37°C for 30 min. The cell culture medium was then removed, and the cells were washed three times with the serum‐free cell culture medium to sufficiently remove DCFH‐DA that did not enter the cells. Then, the laser group was irradiated for 5 min, and the pictures were taken with LSM‐880 immediately after the laser.

#### 3.2.12 Mitochondrial stress detection

Cells were seeded in confocal dishes at a density of 1 × 10^5^, and the sample processing procedure was the same as the confocal experiments in Sections 2.2.8 and 2.2.9. Then, the cell culture medium was removed, washed three times with PBS, and then 1 mM Mito‐Tracker Green stock solution was added to the cell culture medium at 1:10,000 and incubated with cells at 37°C for 45 min. Subsequent observations were made with LSM800.

#### 3.2.13 Western blotting assay

HT‐29 cells were seeded in the 6‐well plates with 5 × 10^5^ cells per well and incubated overnight and then treated with free drugs and the NPs with the equivalent concentrations of cisplatin of 8 µg/ml, respectively, for 24 h. Then, the laser group removed the drug‐containing medium, added fresh medium, and irradiated the laser. Then, the medium is continued to cultivate for 24 h. Then the cells were washed three times with PBS and incubated with RIPA buffer, which was added with a certain concentration of protease inhibitors for 2 h. After cell lysis, the supernatant was collected to standard western blotting, which was based on the protocol from Bio‐Rad Company.

#### 3.2.14 Statistical methods

All of the data in this article were analyzed by the Graphpad Prism 6 software and FlowJo software. Statistical differences were followed; Student's t‐test and two‐way ANOVA are determined. Data are expressed as mean ± SD values. *p < 0.05, **p < 0.01, and ***p < 0.001 denote statistically significant differences.

## 4 RESULTS

### 4.1 The preparation and characterization of FA‐NP

**FIGURE 1:**
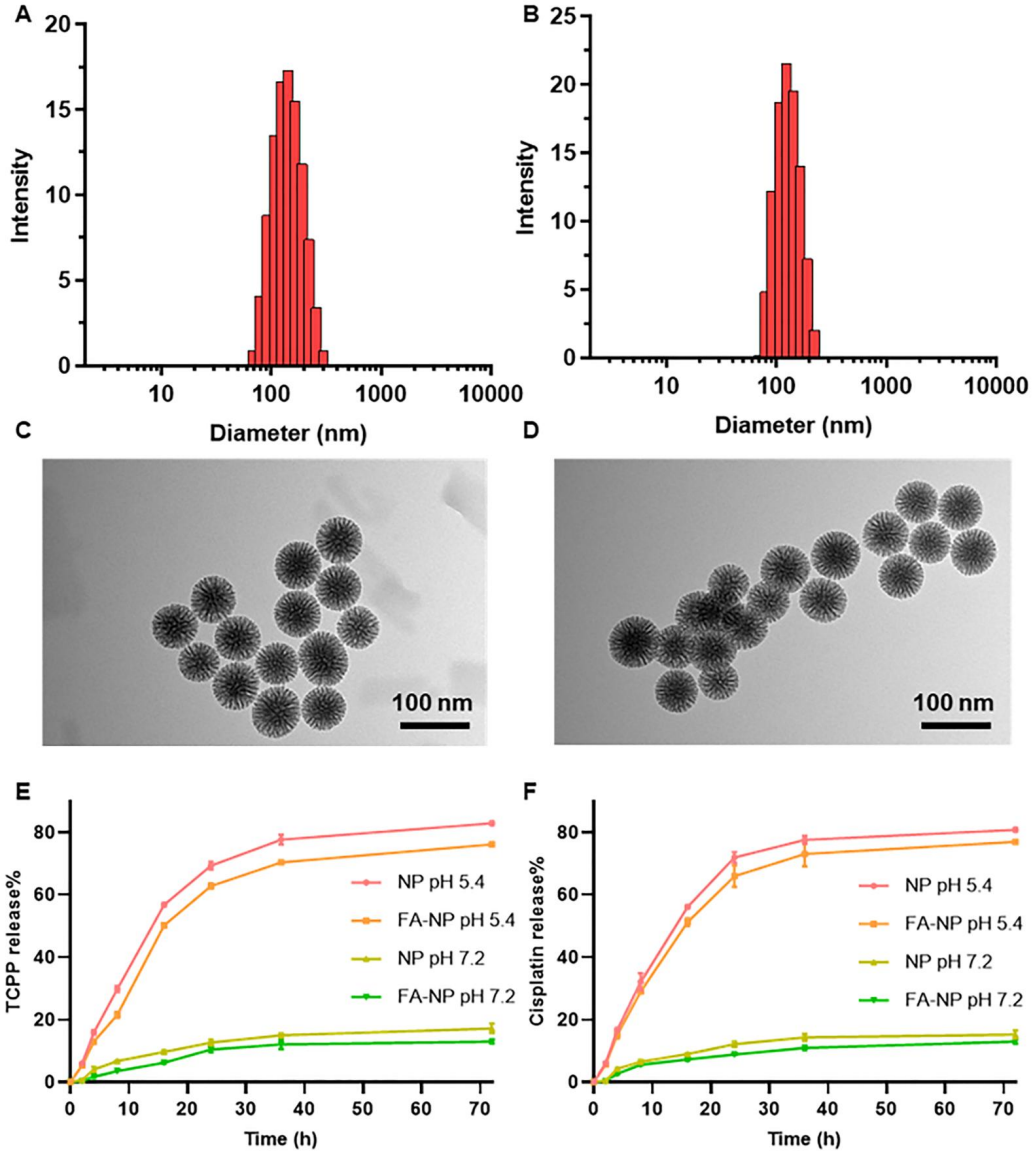
(A) Particle size of the MSN nanoparticles with drug load; (B) Particle size of the FA‐NP nanoparticles with drug load; (C) The TEM of the MSN nanoparticles with drug load; (D) The TEM of the FA‐NP nanoparticles with drug load; (E) Release efficiency of TCPP in different pH values; (F) Release efficiency of cisplatin in different pH values. FA‐NP, folate acid ligand‐modified nanoparticle; MSN, mesoporous silica; TCPP, Tris(2‐chloroisopropyl) phosphate; TEM, tumor microenvironment.

In the study, we first synthesized MSN nanoparticles and detected the size and shape (Figure S2A,B), and then folic acid was modified on the surface of the nanoparticles, followed by drug loading. According to Figure 1A,B and Table S1, the particle size of MSN nanoparticles after drug loading was about 121.8 nm, and the particle size of nanoparticles after folic acid modification increased, and the particle size was about 133.4 nm. All the drug‐loaded NPs have a good particle size distribution and negative ζ‐potential, which is suitable for uptake by cells (Figure S2 and S1). Furthermore, TEM results showed that all NPs are in uniform sizes and are also spherical (Figure 1C,D). We further measured the drug loading. According to Table S1, the drug loading of cisplatin without modification was 14.06% ± 0.17, the encapsulation efficiency was 71.81% ± 0.56, the drug loading of TCPP was 14.45% ± 0.98, and the encapsulation efficiency was 67.95% ± 3.32. After modification of folic acid, although a certain steric hindrance was increased, it did not affect the drug loading. The drug loading efficiency of cisplatin was 11.25% ± 0.35, the encapsulation efficiency was 69.50% ± 1.09, and the drug loading of TCPP was 13.41% ± 0.49. The encapsulation efficiency was 64.01% ± 1.61. Next, to verify the drug release behavior of the nanoparticles, we simulated the pH of normal body fluids (pH = 7.2) and the specific pH of the TEM (pH = 5.4). According to the results, Figure 1E,F shows that both nanoparticles are stable at pH 7.2, and the drug is hardly released, avoiding the toxic and side effects of the drug release on normal tissues. However, when simulating the pH value of the TEM, MSN can be slowly degraded under acidic conditions, so the drug is gradually released with the degradation of MSN. Our results also show that the two nanoparticles have no sudden release behavior within 30 min, which avoids the sudden increase of the blood drug level in the body and causes adverse reactions, and the cumulative drug release reaches more than 75% within 36 h. The drug loading and release pattern of both nanoparticles met the requirements of subsequent experiments.

### 4.2 Cell cytotoxicity and uptake efficiency

**FIGURE 2:**
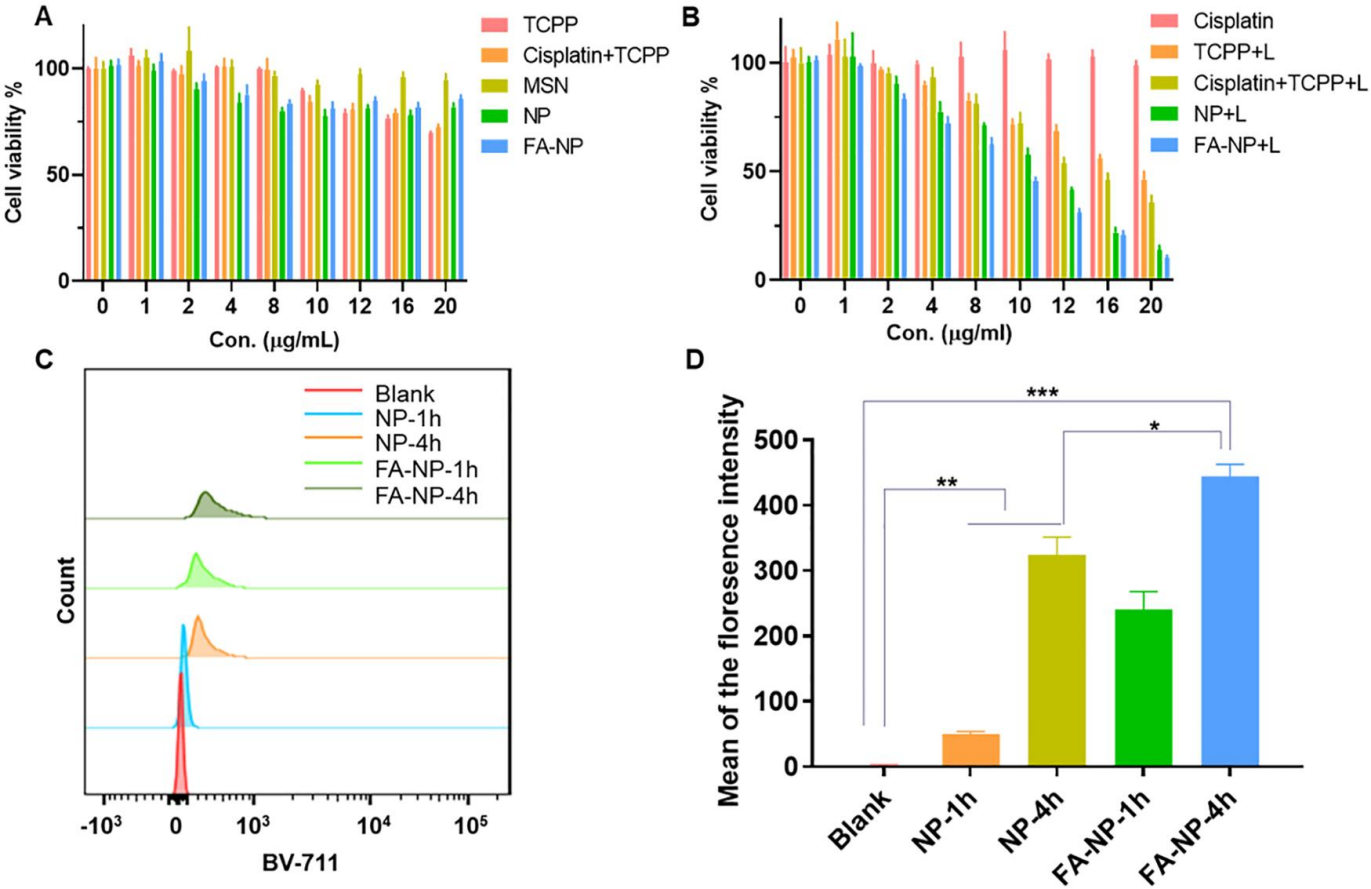
(A) Cell cytotoxicity of different groups without laser; (B) Cell cytotoxicity of different groups with laser; (C) The uptake efficiency of NPs and FA‐NPs at 1 and 4 h; (D) Statistical data of mean fluorescence intensity from NPs and FA‐NPs uptake. FA‐NP, folate acid ligand‐modified nanoparticle.

Next, we verified the toxicity of nanoparticles to healthy cell lines and drug‐resistant colon cancer cells. As shown in Figure S3, free TCPP and cisplatin caused certain cytotoxicity in the MCF‐10A cell line. And we also found that the nanocarrier group (MSN group) did not arouse any cytotoxicity, which proved that MSN has high biocompatibility. Besides, we found that loading the drugs into the nanocarriers can reduce cytotoxicity. It is consistent with the result in Figure 1E,F that in the physiologic pH value, the drugs will not release from nanocarriers, so it does not matter without laser or with laser; NP and FA‐NP groups did not cause the cytotoxicity, which show that the nanoformula can protect the healthy cells from killing. In Figure 2A,B, HT‐29 cells were able to completely resist the cytotoxic effect of cisplatin. In addition, the nanocarriers themselves did not lead to cell death, and the drug‐loaded nanoparticles did not cause obvious cytotoxicity without laser irradiation, indicating that the cascade reaction triggered by the laser is the key to causing cell death. Therefore, both free TCPP and TCPP‐containing nanoparticles produced different degrees of cytotoxicity on HT‐29 cells after laser irradiation. It can be seen from Table S2 that the IC_50_ value of the TCPP plus laser group was 18.34 µg/ml, and the IC_50_ value of the nanoparticle group with or without folic acid ligand was significantly lower than that of the free drug group, which were 10.35 µg/ml and 8.139 µg/ml, respectively, showing stronger cell cytotoxicity effect. The results showed that the nanoparticle group with the target ligand had the best effect. This is related to the targeting of the folate receptor, which can mediate the accumulation of nanoparticles at the tumor site for a specific time.

Therefore, we next verified the uptake efficiency of nanoparticles by HT‐29 cells. The results from Figure 2C,D showed that within 4 h, compared with unmodified nanoparticles, folic acid‐modified nanoparticles are more easily taken up by tumor cells, because the modified nanoparticles can not only enter cells relying on phagocytosis but also use receptor‐mediated endocytosis into cells. In addition, the high expression of folate receptors on the surface of tumor cells increases the characteristics of targeting, which can not only improve the affinity with tumor cells but also effectively reduce the toxic side effects caused by healthy cell uptake.

### 4.3 FA‐NPs trigger immunogenic cell death

There are many ways to enhance the immunogenicity of tumor cells; one of them is to trigger tumor cells to transfer from nonimmunogenic to immunogenic when they are not specifically killed. In this way, it can mediate antitumor immune responses, which are also called immunogenic cell death (ICD).^21^ It is known that when tumor cells are under the control of ICD, they recruit various related signaling molecules, one of which is called damage‐associated molecular pattern (DAMP), mainly including calreticulin (CRT). Calreticulin will expose on the cell surface when cancer happens to ICD. At the same time, high mobility group protein 1 (HMGB1) will be released outside by cells.^22^, ^23^ Many studies have shown that both chemotherapeutic drugs and photodynamic therapy (PDT) can trigger the tumorigenesis of ICD.

**FIGURE 3:**
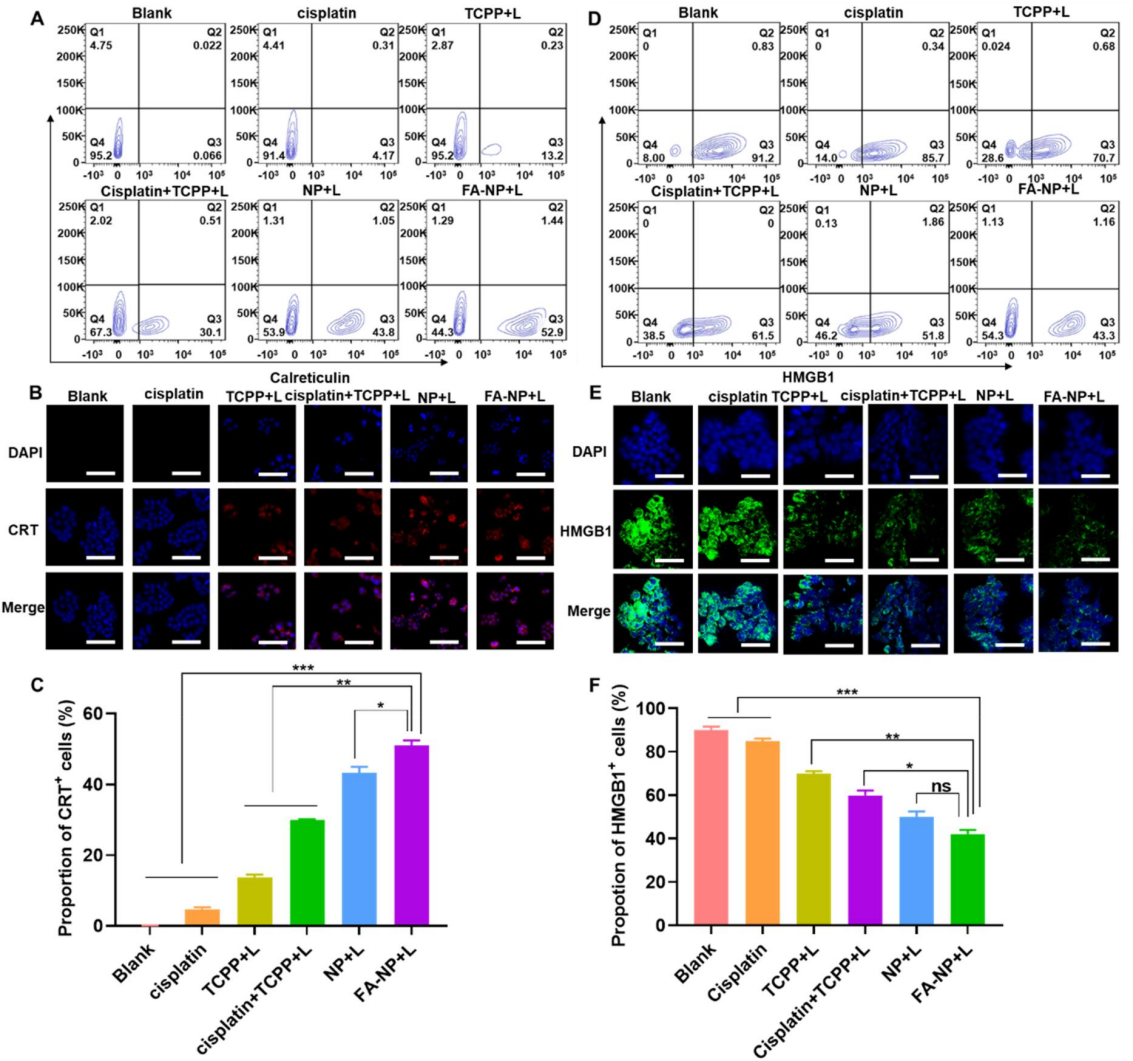
(A) Flow cytometry results of CRT translocated in different groups with laser; (B) Confocal microscopy results of CRT translocated in different groups with laser (scale bar: 100 µm); (C) Statistical data of flow cytometry results of CRT translocated in different groups with laser; (D) Flow cytometry results of HMGB‐1 release in different groups with laser; (E) Confocal microscopy results of HMGB‐1 release in different groups with laser (scale bar: 100 µm); (F) Statistical data of flow cytometry results of HMGB‐1 release in different groups with laser. CRT, calreticulin.

Therefore, we further verified whether FA‐NPs could induce significant ICD responses in tumor cells. Figure S4A,B demonstrated that the free cisplatin and TCPP and the nanoparticle group without laser did not significantly cause the CRT of HT‐29 cells to transfer from the inner membrane to the outer membrane, while the group, given the laser (Figure 3A,B), free TCPP + L could cause 13.2% of the CRT translocated, while the free drug group combined with cisplatin (Cisplatin + TCPP + L) could cause 30.1% of the CRT translocated, and the NP + L group could cause 43.8% of the CRT translocated, while the FA‐NP + L group could induce 52.9% CRT translocated.

In addition, according to Figure S4C,D, HT‐29 cells highly expressed HMGB‐1 protein, and the experimental groups using cisplatin and without laser did not stimulate the release of HMGB‐1 outside the cells, but after adding laser (Figure 3D,E), the TCPP + L group can induce about 20% of HMGB‐1 to be released into the extracellular, the combination group (Cisplatin + TCPP + L) can induce about 30% of the release, and the nanoparticle group can further stimulate the release of HMGB‐1 into the extracellular after adding laser, respectively, around 40% and around 50%. All the statistical results are shown in Figure 3E,F. The combination of TCPP can promote the translocation of CRT and the release of HMGB1 under laser stimulation, and the effect of the nanosystem is better than that of the free drug group. Here, we demonstrate that the FA‐NP group can significantly induce the transfer of CRT to the outer membrane under the stimulation of laser, promote the release of HMGB‐1 into the extracellular, induce cells to produce ICD, and then trigger cell death.

### 4.4 Combined with photodynamic enhancement of intracranial ROS

Certain concentrations of ROS play an irreplaceable role as signaling molecules in cells. High concentrations of ROS often induce apoptosis, but high concentrations of ROS are precisely one of the characteristics of drug‐resistant cancer cells. Higher levels of ROS in drug‐resistant cancer cells, including those from cancer patients, lead to higher levels of antioxidant enzymes; this trait is distinguishing them from other cells. Therefore, further increasing its ROS level or inhibiting its antioxidant enzymes can selectively kill drug‐resistant cancer cells.^8^, ^24^ PDT can activate photosensitizers in tumor sites to generate biologically toxic singlet oxygen and other ROS by irradiating specific wavelengths of light sources.

**FIGURE 4:**
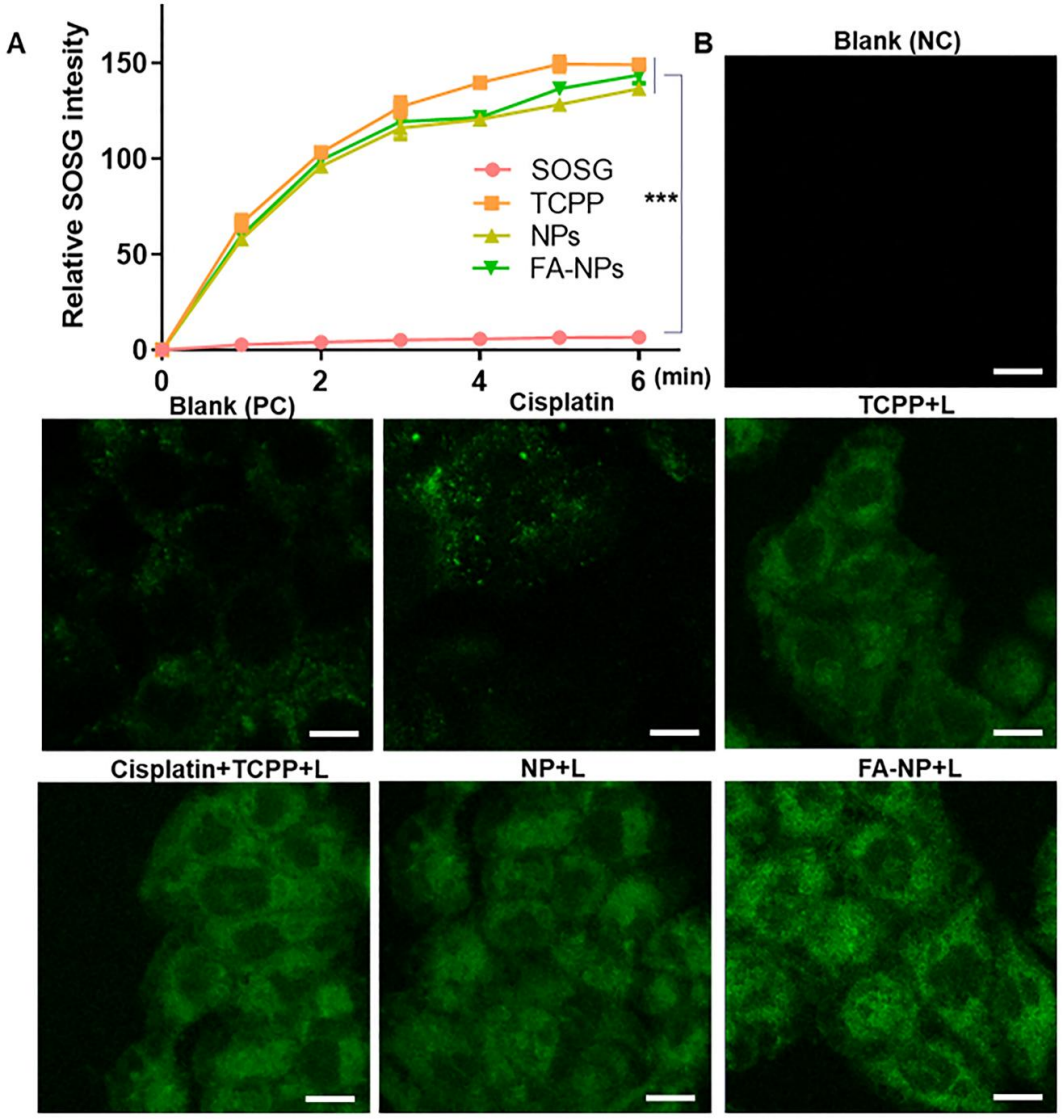
(A) NPs and FA‐NPs trigger the singlet oxygen generation; (B) Intracellular ROS level with different administration (with laser; scale bar: 100 µm). FA‐NP, folate acid ligand‐modified nanoparticle; ROS, reactive oxygen species.

In this study, TCPP was used as a photosensitizer. First, we demonstrated that under laser stimulation, TCPP and the nanoparticle groups can generate singlet oxygen and react with SOSG to generate green fluorescence. The nanosystem does not affect the photosensitivity of TCPP (Figure 4A). In the cells, as shown in Figures 4B and S5, the drug‐resistant HT‐29 cells contained a certain concentration of ROS, and free cisplatin could not cause an increase in ROS, but regardless of the free TCPP or the combined treatment group, in the treatment group only after laser stimulation, the intracellular ROS content was further increased. Therefore, we know that in drug‐resistant cells, combined photodynamics to disrupt the homeostasis of intracellular ROS can inhibit tumor growth.

### 4.5 Regulation of mitochondria and signaling pathways

**FIGURE 5:**
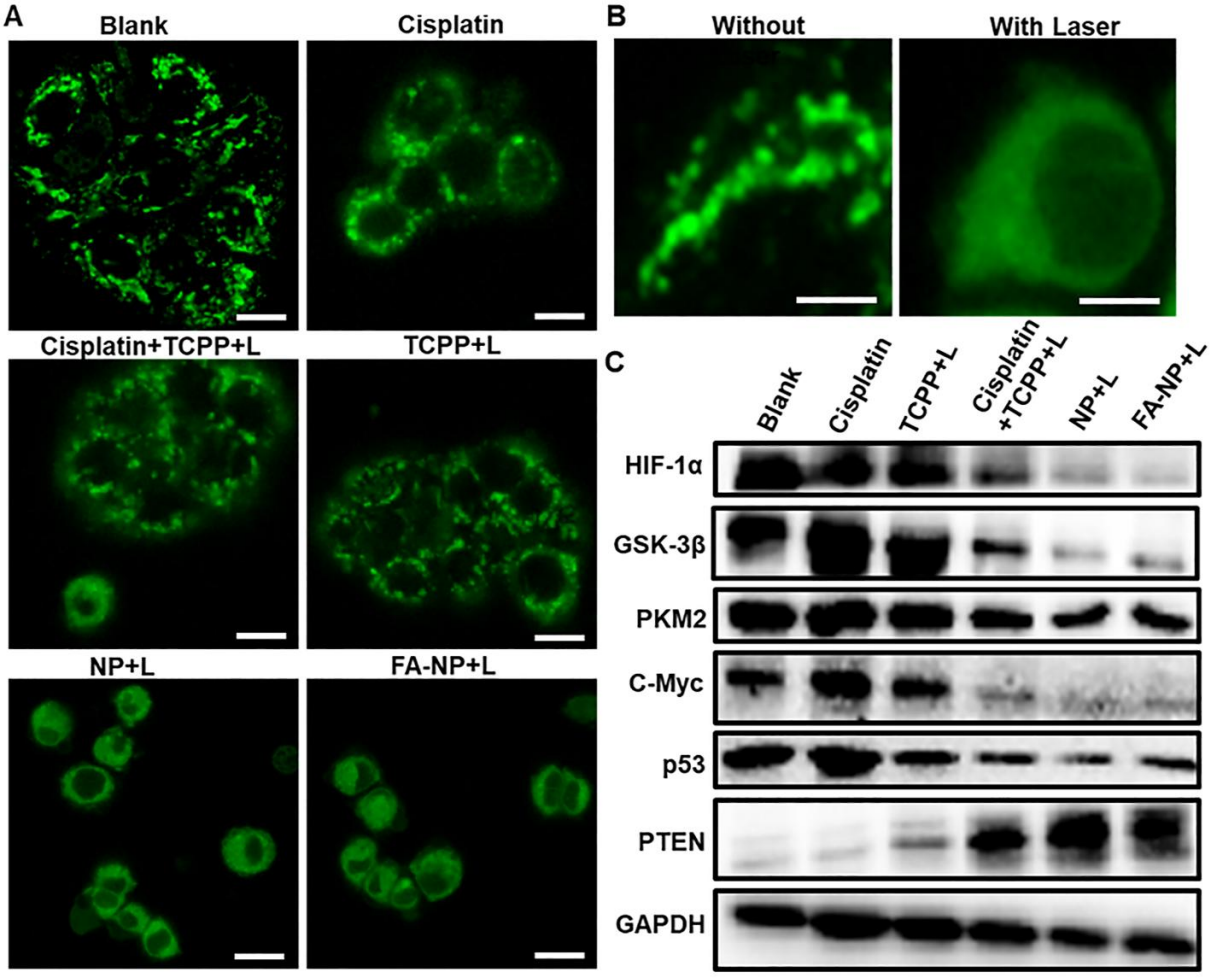
(A) Morphological changes of mitochondria under different administration (with laser; scale bar: 100 µm); (B) Magnified view of mitochondrial morphology without and with laser (scale bar: 200 µm); (C) Western blotting results of different protein expressions.

From the above that triggers the TCPP to further increase the level of intracellular ROS to breaking the ROS homeostasis in the drug‐resistant cell HT‐29, then excess ROS can also induce the breaking of mitochondrial outer membrane pores, resulting in changes in the mitochondrial function. Mitochondria is the foundry of energy, where all life activities need to rely on its energy supply. Therefore, disrupting the homeostasis of mitochondria in tumors has a positive effect on tumor suppression. As shown in Figure 5A,B, in HT‐29 cells, the mitochondrial morphology was line‐like. Even after the treatment with cisplatin and each group without laser (Figure S6), the mitochondria morphology remained the same, but after the addition of laser, the mitochondrial morphology changed from line‐like to dot‐like.

HIF‐1α is an important transcription factor that closely relates to hypoxia, and also, HIF‐1α is involved in many signaling pathway regulations, such as angiogenesis, cell metabolism, apoptosis, etc. The tumor can also promote HIF‐1α activation when the metabolic demands of rapid tumor growth exceed the limits of vascular supply. This condition creates a hypoxic microenvironment in and around the tumor cell mass, which leads to HIF‐1 activation. As mentioned earlier, hypoxia also plays an important role to regulate drug resistance‐related factors. In addition, GSK‐3β is an evolutionarily conserved serine/threonine kinase. GSK‐3β can also regulate the ratio of Bax/HKII by affecting the concentration of glucose in the blood, thereby affecting mitochondrial permeability and cytochrome C release, which is involved in the regulation of apoptosis.^25^ PKM2 is a specific enzyme in cancer tissue and is highly expressed in many cancer cells. It is a restriction enzyme of aerobic glycolysis, which leads to tumor occurrence, development, and metastasis.^26^ The protein is closely related to tumor metabolism. According to western blotting results (Figure 5C), the protein expression levels of HIF‐1α, GSK‐3β, and PKM2 proteins were downregulated, indicating that the microenvironment of tumor hypoxia and abnormal metabolism was improved after administration.

Furthermore, we found that the expression of p53 protein, which is closely related to drug resistance, was also downregulated, while the expression of PTEN protein was upregulated, which reversed the tumor drug resistance microenvironment.^27^ Moreover, C‐myc, a protein closely related to cell proliferation, can enable cells to proliferate indefinitely, obtain immortalization functions, and promote cell division. After administration, the expression of C‐myc was inhibited and tumor cell development was inhibited.

Collectively, our results suggest that the FA‐NP system can mediate mitochondrial stress, thereby regulating a series of related signaling pathways, changing the drug‐resistant microenvironment, and enabling cisplatin to regain its antitumor effect.

## 5 CONCLUSION AND DISCUSSION

This study found that breaking the mitochondrial homeostasis, and then breaking the tumor drug resistance microenvironment, can reverse the drug resistance of chemotherapy drugs. Mitochondria is a popular target, but without any drugs that can directly target mitochondria, more strategies aimed to regulate mitochondrial function. Above all, the results have shown that mitochondria play a vital role in regulating ROS and HIF‐1α. Therefore, when ROS release is triggered by TCPP, breaking the balance of ROS in the microenvironment, we observed that mitochondria morphology changed and the expression of metabolism‐related proteins regulated by mitochondria changed correspondingly. In turn, the expression of p53, a protein directly related to drug resistance, was directly inhibited. A series of modulations finally restored the function of cisplatin and inhibited the growth of CRC cells. In future studies, we plan to be able to benefit from more resistant cell lines and hopefully test their effects in vivo. We are looking forward to bringing a new hope to the treatment of drug‐resistant tumors.

## AUTHOR CONTRIBUTIONS

Yonghui Wang: Data curation; Formal analysis; Investigation; Writing original draft. Xiaodong Ma: Visualization; Methodology; Validation. Wenhui Zhou: Visualization; Methodology; Validation. Chang Liu: Visualization; Methodology; Validation. Hongbo Zhang: Formal analysis; Project administration; Supervision; Writing – review & editing.

## CONFLICT OF INTEREST

The authors declare that they have no competing interests. Hongbo Zhang is a member of the *Smart Medicine* editorial board.

## ETHICS STATEMENT

This article does not contain any studies involving animal and human participants performed by any of the authors.

**Abbreviations**
CRC: colorectal cancer
DL: drug‐loading capacity
FA: folate acid
FA‐NPs: folate acid nanoparticles
HIF‐1a: hypoxia‐inducible factor‐1 alpha
ICD: immunogenic cell death
MDR: multidrug resistance
ROS: reactive oxygen species
SO: super oxide
TCPP: Tris(2‐chloroisopropyl) phosphate
TEM: transmission electron microscope

## Supporting information

Supporting Information S1
