# Correction to “Reregulated Mitochondrial Dysfunction Reverses Cisplatin Resistance Microenvironment in Colorectal Cancer”

**DOI:** 10.1002/smmd.70001

**Published:** 2025-02-25

Y. Wang, X. Ma, W. Zhou, C. Liu, H. Zhang, Reregulated mitochondrial dysfunction reverses cisplatin resistance microenvironment in colorectal cancer. *Smart Med*. 2022, 1(1), e20220013.

In this article, the full name of TCPP should be Tetra(4‐carboxyphenyl)porphyrin, but the article incorrectly refers to it as Tris(2‐chloroisopropyl) phosphate in several instances. This error appears in four locations:

1. The abstract on the first page
2. The list of abbreviations on the first page
3. The final part of the introduction on the third page
4. The caption of Figure 1 on the sixth page

We apologize for this error.

