# Supplementary material for: Reregulated mitochondrial dysfunction reverses cisplatin resistance microenvironment in colorectal cancer
Source: Smart Med. 2022 Dec 22;1(1):e20220013. doi: 10.1002/SMMD.20220013 (PMC11235731; doi:10.1002/SMMD.20220013)
Supplement: Supplementary file 1 — Supporting Information S1 [file SMMD-1-e20220013-s001.docx]

**Supporting Information**

**Reregulated mitochondrial dysfunction reverses cisplatin resistance microenvironment in colorectal cancer**

Yonghui Wang^1,2^, Xiaodong Ma^1,2^, Chang Liu^1,2^, Wenhui Zhou^1,2^, Hongbo Zhang^1,2*^

^1^Pharmaceutical Sciences Laboratory,Åbo Akademi University, Turku 20520,Finland

^2^Turku Bioscience Centre, University ofTurku and Åbo Akademi University,Turku 20520, Finland

E-mail: hongbo.zhang@abo.fi

*Corresponding Author


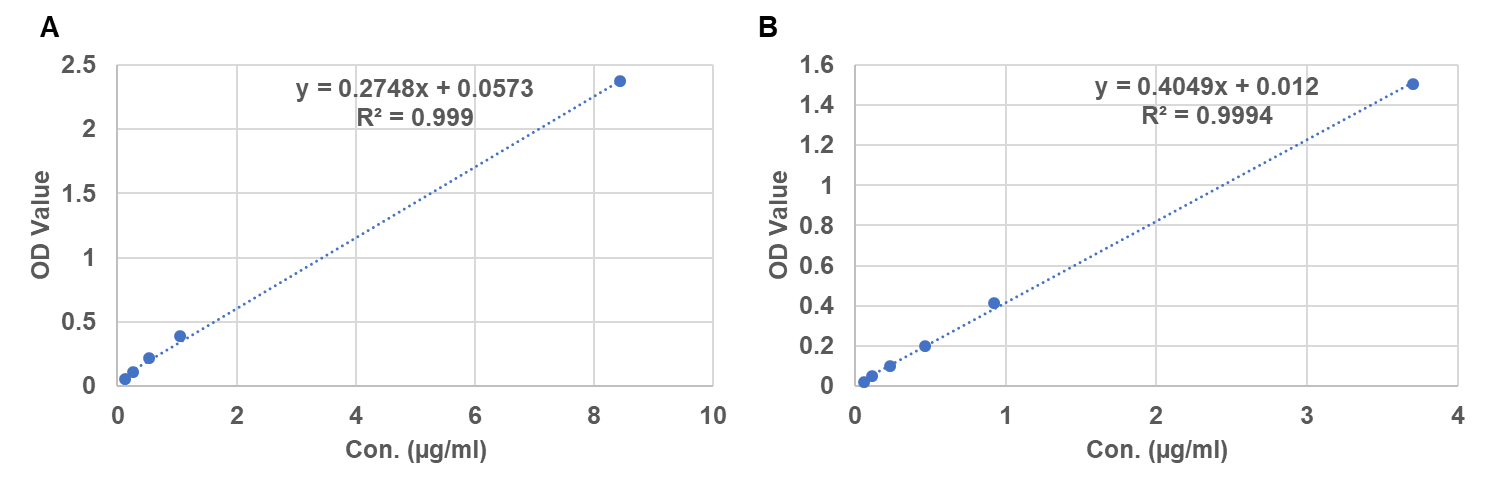


**Figure S1:** A: Standard curve of TCPP; B: Standard curve of cisplatin


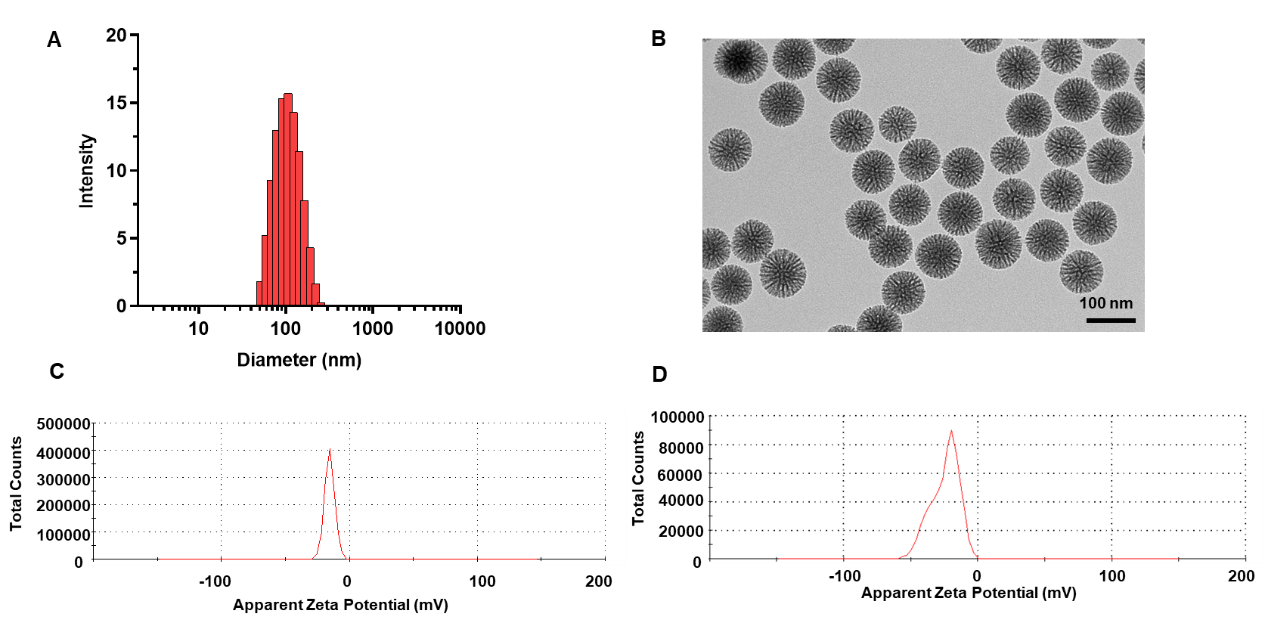


**Figure S2:** A: Particle size of the MSN nanoparticles without drug load; B: The TEM of the MSN nanoparticles without drug load; C: Zeta potential of the NPs; D: Zeta potential of the FA-NPs


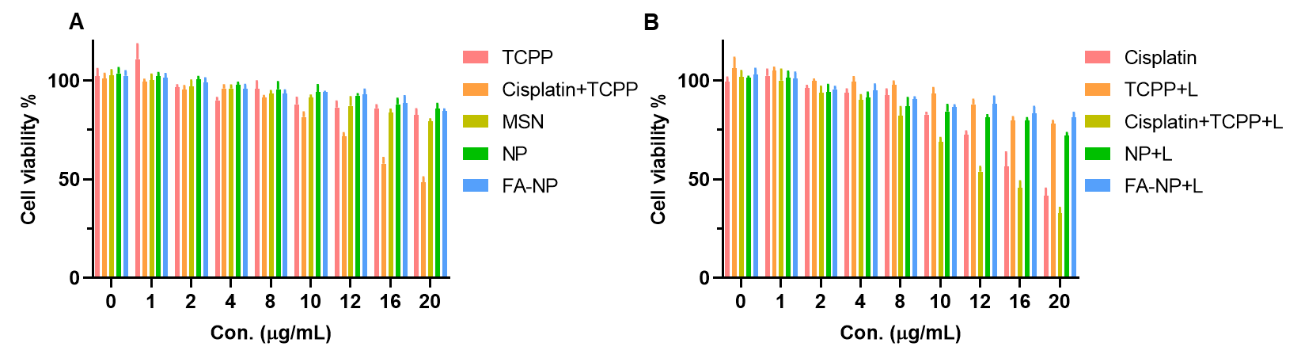


**Figure S3:** Cell cytotoxicity in healthy cell line (MCF-10A). A: different groups without laser; B: different groups with laser.

**Table S1:** The characteristics of NPs and FA-NPs


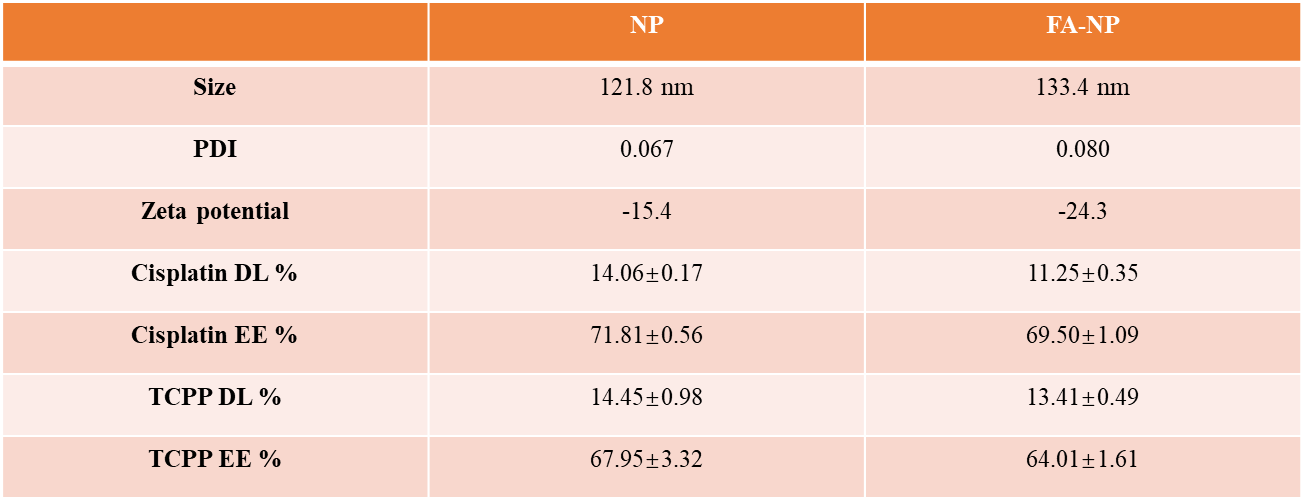
 **Table S2:** The IC_50_ of different groups


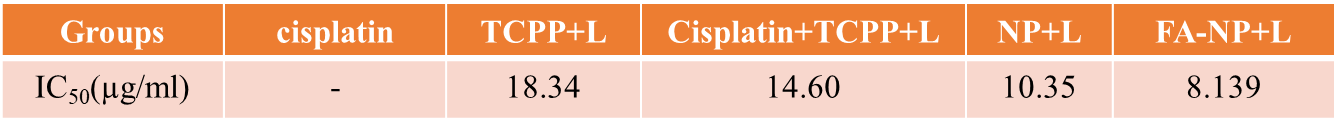


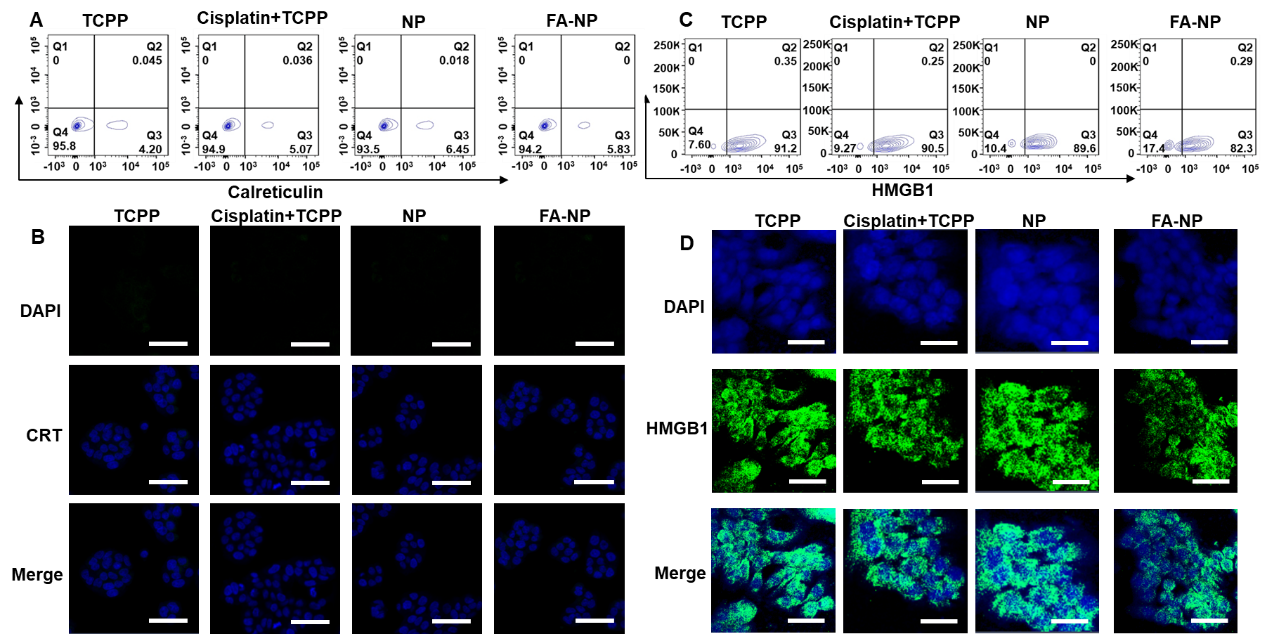


**Figure S4:** A: Flow cytometry results of CRT translocated in different groups without laser; B: Confocal microscopy results of CRT translocated in different groups without laser (scale bar: 100 µm); C: Flow cytometry results of HMGB-1 release in different groups without laser; D: Confocal microscopy results of HMGB-1 release in different groups without laser (scale bar: 100 µm)


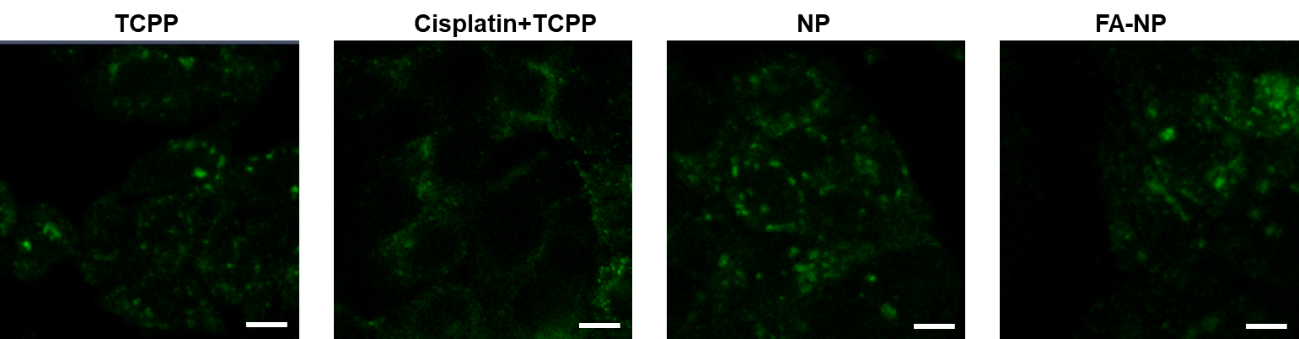


**Figure S5:** Intracellular ROS level with different administration (without laser; scale bar: 100 µm)


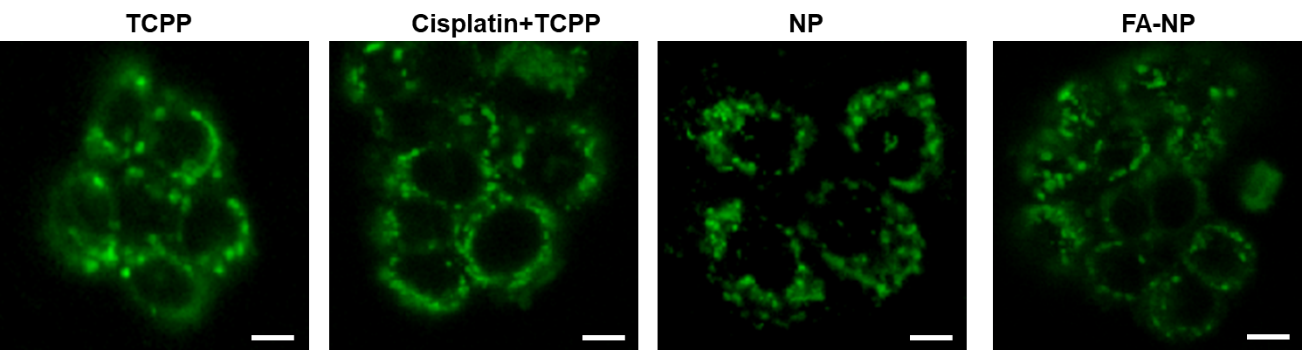


**Figure S6:** Morphological changes of mitochondria under different administration (without laser; scale bar: 100 µm)
